# Preparation, Characterization, and Biological Properties of Hydroxyapatite from Bigeye Snapper (*Priancanthus tayenus*) Bone

**DOI:** 10.3390/ijms24032776

**Published:** 2023-02-01

**Authors:** Nunnuth Jindapon, Phatthranit Klinmalai, Utoomporn Surayot, Nuttapol Tanadchangsaeng, Woradej Pichaiaukrit, Yuthana Phimolsiripol, Chaluntorn Vichasilp, Sutee Wangtueai

**Affiliations:** 1 College of Maritime Studies and Management, Chiang Mai University, Samut Sakhon 74000, Thailand; 2 College of Biomedical Engineering, Rangsit University, Pathumthani 12000, Thailand; 3 College of Dental Medicine, Rangsit University, Pathumthani 12000, Thailand; 4 Faculty of Agro-Industry, Chiang Mai University, Chiang Mai 50100, Thailand; 5 Faculty of Natural Resources, Rajamangala University of Technology Isan, Sakon Nakhon 47160, Thailand

**Keywords:** hydroxyapatite, bigeye snapper, *Priancanthus tayenus*, fish bone, protein adsorption, protein desorption

## Abstract

The optimum condition of acid hydrolysis for hydroxyapatite extraction from bigeye snapper (*Priancanthus tayenus*) bone and the effects of extraction time (10–60 min) and HCl concentration (2.0–5.0% *w*/*v*) on yield and hydroxyapatite properties were determined. The optimum extracted condition was found using 5% HCl for 60 min, which was 13.4% yield; 19.8 g/100 g Ca content; 9.6 g/100 g P content; 2.1 Ca/P ratio; L*, a*, b*; and Δ*E* as 84.5, 2.8, 16.5, and 15.6, respectively. The using of 5% NaOH solution was optimum for hydroxyapatite precipitation from the extracted solution. The characteristic and biological properties of the obtained hydroxyapatite were studied. Fourier transform infrared spectroscopy and X-ray diffraction results showed a good comparison between the extracted and commercial hydroxyapatite. The microstructure of the extracted hydroxyapatite from a scanning electron microscope showed an irregular and flat-plate shape, large surface area, and roughness. The extracted hydroxyapatite was non- and low-cytotoxicity at a concentration of 50 and 100–400 µg/mL, respectively. Bovine serum albumin (BSA) adsorption and desorption of hydroxyapatite was studied. An increasing BSA concentration, hydroxyapatite amount, and adsorption time significantly increased protein adsorption on hydroxyapatite. Protein desorption from BSA-loaded hydroxyapatite showed an increase of release initially in the first 4 days and became a steady release rate until 14 days.

## 1. Introduction

The expansion of fish processing has generated increasing quantities of byproducts that may account for up to 70% of the whole fish weight, depending on the raw materials and finished products. In general, the fish byproducts are composed of 9–12% heads, 12–18% viscera, 1–3% skins, 9–15% bones, and ~5% scales in the total fish weight [1]. For example, a by-product from surimi or fish mince production may account ~60–70% of the whole fish weight, including the head, viscera, skin, bone, scale, fin, etc. [2]. Historically, the fish byproducts were often used for fish meal and oil production or discarded as waste, resulting in underutilized fish resources and economic losses, as well as environmental effects [1]. Eventually, fish by-product utilization gained increasing attention. It is often involved used for developing extracted ingredients, bioactive compounds, or value-added products in various forms based on potential valid alternatives of fish by-products. In addition, due to the high content of collagen, enzymes, peptides, polyunsaturated fatty acids, and minerals in fish by-products, they also provide a large and sustainable supply of high-value bio-compounds that are used in biotechnological, medicinal, and pharmaceutical applications [3]. Especially, fish bones have been used as a source of protein and mineral extraction such as collagen, gelatin, calcium, bio-calcium, and calcium phosphate or hydroxyapatite [4]; for example, the bones of bigeye snapper (*Priacanthus tayenus*) are a good source for collagen, gelatin, and gelatin hydrolysate preparation [5,6]. 

Hydroxyapatite is a calcium phosphate compound, Ca_10_(PO_4_)_6_(OH)_2_, that serves as the main inorganic element composition in the bone and teeth [7]. Hydroxyapatite is also generally recognized as a biomaterial used for medical applications in bone and dental treatments. Normally, hydroxyapatite can be obtained using two major processes including synthesis from chemicals and extraction from biological resources [8]. The obtained hydroxyapatite from animal bones has a few advantages over the synthetic types such as chemical composition and structure that it is similar to that of human bone, lower processing cost, and improved biological response [9]. Natural resources have been used for hydroxyapatite extraction such as bovine bones [10], pig bones [11], and eggshells [12]. However, the hydroxyapatite produced from mammalian bone might have been limited for reasons of biosafety and religion. More attention is probably given to aquatic animal resources as a material for hydroxyapatite production [13]. Various works have studied hydroxyapatite preparation from fish such as the bone of salmon, rainbow trout, cod, catfish, tilapia, seabass, yellowfin tuna, and Whitemouth croaker [8,14,15,16]. 

Hydroxyapatite extracted from fish bone is considered to be an alternative to synthetic hydroxyapatite from chemicals. It has recently been recognized as a promising biomaterial with great potential in biocompatibility and bioactivity, and its inorganic composition is similar to that of natural bone [4,15]. Hydroxyapatite has been used as an adsorbent in chromatography for isolation or purification because the surface itself has a high adsorption ability for many substances [17]. The biological characteristics of calcium phosphate or hydroxyapatite biomaterials are mostly determined by protein adsorption and desorption properties [18]. Hydroxyapatite has been studied in protein adsorption for carriers of protein drugs. The protein adsorption and release properties of hydroxyapatite might be related to the surface properties that are also affected by the preparation process [17]. Previous reports have studied the biological properties of hydroxyapatite for protein adsorption and desorption by testing with several proteins such as bovine serum albumin (BSA), lysozyme, cytochrome c, insulin, myoglobin, and ovotransferrin [7,19,20,21]. 

This study aimed to prepare hydroxyapatite from bigeye snapper bone, a byproduct from the surimi or fish mince processing, using the acid extraction methods. The optimum extracted condition and mathematical model for hydroxyapatite extracted from bigeye snapper bone were generated, and the characterization and functional properties of the obtained hydroxyapatite powder were determined. In addition, the biological properties of protein adsorption and desorption, and cell cytotoxicity were also carried out to demonstrate in vitro biocompatibility.

## 2. Results and Discussion

### 2.1. Chemical Composition of Bigeye Snapper Bone

Fresh bigeye snapper bone contained 57.27% moisture, 16.13% protein, 9.04% fat, and 16.80% ash. This result was consistent with the study of Kittiphattanabawon et al. [5], in which the content of moisture, protein, fat, and ash were 62.27, 13.3, 8.77%, and 14.40% ash content, respectively. While Jaziri et al. [22] reported that bigeye snapper bone had a content of 71.31% moisture, 12.46% protein, 1.29% fat, and 13.97% ash. However, in previous studies and present work, the bigeye snapper bone contained high ash content (ranging about 14–17%), and then it was possibly used as a material to obtain the hydroxyapatite.

### 2.2. Optimization of Hydroxyapatite Extraction Condition

RSM with a faced-centered central composite design was performed. The effects of the extraction time (X_1_: 10–60 min) and the concentration of HCl (X_2_: 2–5% *w*/*v*) on yield and hydroxyapatite properties (Ca and P content, Ca/P ratio, and color parameters) were determined as shown in Table 1. Regression analysis was carried out to develop the response surface models with full quadratic models as shown in Table 2. Most of the models were significant (*p* ≤ 0.05), except for the models of Ca and P contents and Ca/P ratio which were not significant (*p* > 0.05). The coefficients of determination (R^2^) of all models were in the range of 0.47–0.98. The appropriated models for indicated correlations among the variable parameters with high confidence have to be significant in the model (lower *p*-value) and higher R^2^ then to explain the effects of the variables in the experimental data [23,24].

The multi-responses optimization was performed using five responses of yield, L*, a*, b*, and Δ*E* values. The optimum extraction time (X_1_) and HCl concentration (X_2_) to obtain the highest yield and good color were 60 min and 5% *w*/*v*, respectively. The value criteria of parameters for multi-response optimization, optimum condition, composite desirability, predicted values, and validated experimental values are shown in Table 3. The experimental values were not significant with the predicted values (*p* > 0.05) for all responses, showing good predictor models. Based on its higher yield and better color, the hydroxyapatite was prepared using a condition of 5% *w*/*v* HCl solution and extraction for 60 min.

**Figure 1:**
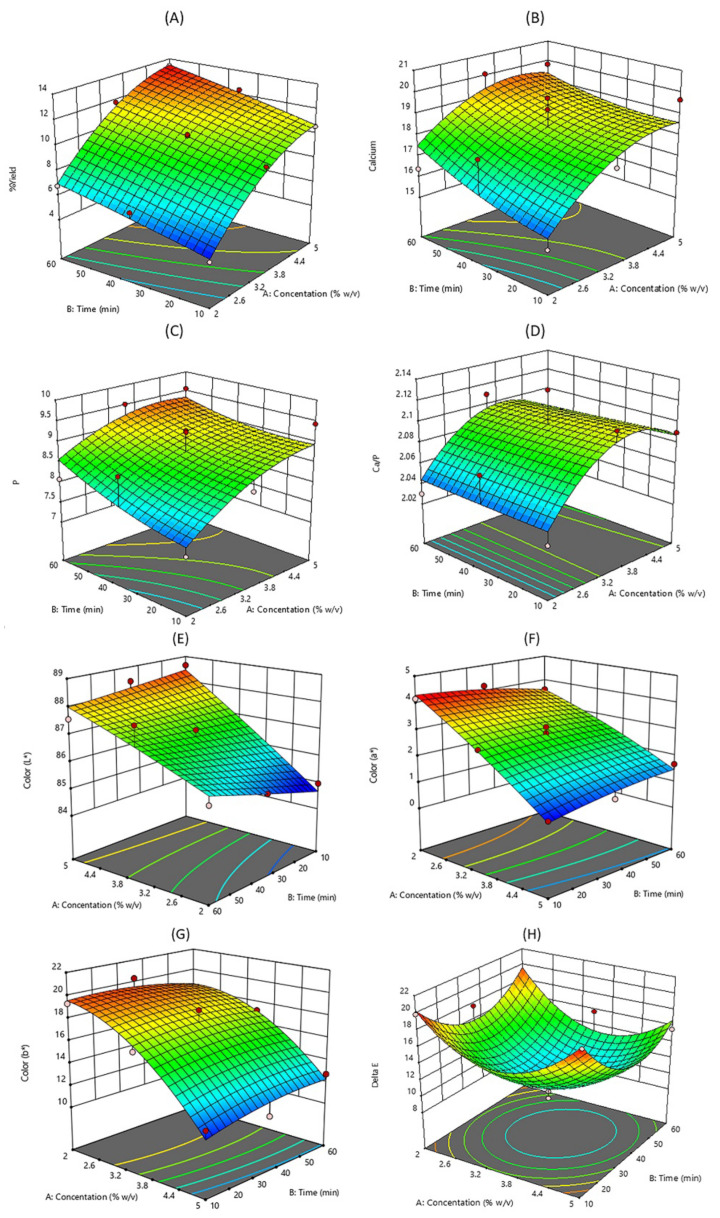
Response surface plots showing the effect of extraction time and HCl concentration on yield (**A**), Ca (**B**) and P (**C**) contents, Ca/P ratio (**D**), and color properties (**E**–**H**).

The effects of extraction time (X_1_) and HCl concentration (X_2_) on responses are shown in Figure 1. Both X_1_ and X_2_ were significant main effects without interactions effect on the hydroxyapatite yield. In Figure 1A, the yield increased significantly with extraction time and HCl concentration. This might be due to a higher acid concentration and increase in contact time with higher dissociation of hydroxyapatite from the matrix in the fish bone to the solution [25]. Then, a higher yield was obtained after precipitation by a NaOH solution. Muhammad et al. [26] reported that hydroxyapatite extraction from bones and fish scales clouds involved using acids, alkalis, and heat treatments to further separate the hydroxyapatite from biological resources. For the calcium and phosphorus contents and Ca/P ratio, the X_1_ and X_2_ were not significant main effects and were without interaction effects. The increasing Ca and P contents with increasing HCl concentration and extraction time were observed (Figure 1B,C), while the highest Ca/P ratio was found when the HCl concentration was about 3.5% *w*/*v* (Figure 1D). For the response of color parameters, the X_2_ significantly influenced L*, a*, and b* values. The higher HCl concentration resulted in higher L*, a*, and b* values (Figure 1E–G), and the lowest Δ*E* was observed at about the center level of both X_1_ and X_2_ (3.5% HCl concentration and 30 min extraction time) as shown in Figure 1H.

### 2.3. Characterization of Extracted Hydroxyapatite

**Figure 2:**
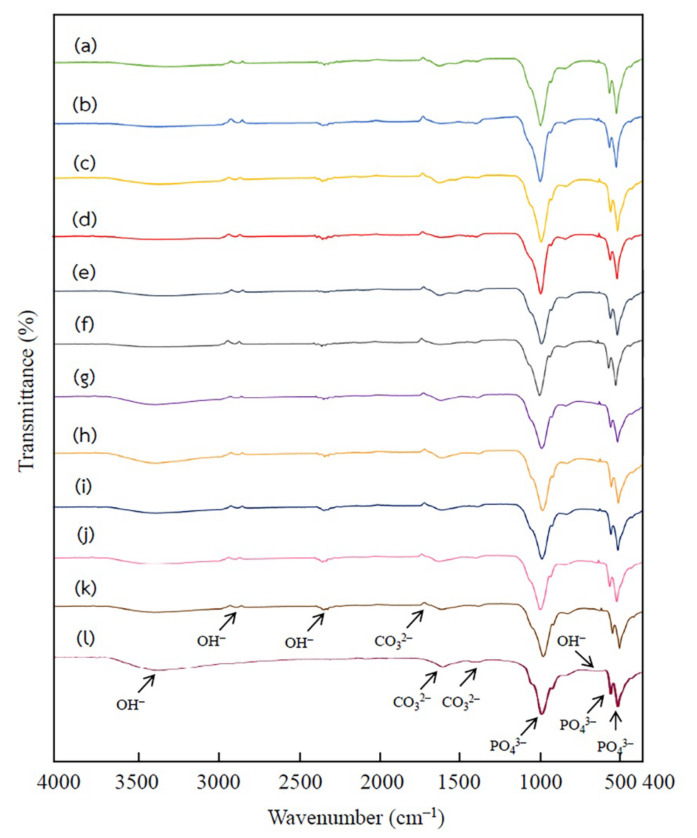
The FTIR spectra of hydroxyapatite extracted using 2% HCl 10 min (**a**), 5% HCl 10 min (**b**), 2% HCl 60 min (**c**), 5% HCl 60 min (**d**), 2% HCl 35 min (**e**), 5% HCl 35 min (**f**), 3.5% HCl 10 min (**g**), 3.5% HCl 60 min (**h**), 3.5% HCl 35 min Rep.1 (**i**), 3.5% HCl 35 min Rep.2 (**j**), 3.5% HCl 35 min Rep.3 (**k**), and the commercial hydroxyapatite (**l**).

The FTIR spectra from all 11 treatments and commercial hydroxyapatite (Sigma-Aldrich) are presented in Figure 2. All hydroxyapatite showed a series of usual characteristic bands for phosphate (PO_4_^3−^), carbonate (CO_3_^2−^), and hydroxyl ions (OH^−^). In Figure 2, the peaks at 595, 870–874, and 1024 cm^−1^ are attributed to the bending vibration of PO_4_^3−^, a stretching vibration of P–O bonds [27,28]. The bands at 1649 and 1747 cm^−1^ indicated the formation of the CO_3_^2−^ [29,30]. The CO_3_^2−^ is also found at bands ranging from 1412–1455 cm^−1^. This was in accordance with the report of Ebrahimi et al. [31] (2021) that the CO_3_^2−^ band appeared at 1430 cm^−1^ because of the formation of CO_2_ in the atmosphere and sufficient hydroxyl in the alkali conditions. In addition, the peaks ranging from 660–675 cm^−1^, 2347 cm^−1^, 2854–2929 cm^−1^, and 3370–3404 cm^−1^ showed the adhesion and bending of water molecules referring to the hydroxyl group (OH^−^), and the presence of adsorbed water in the structure [30,32].

**Figure 3:**
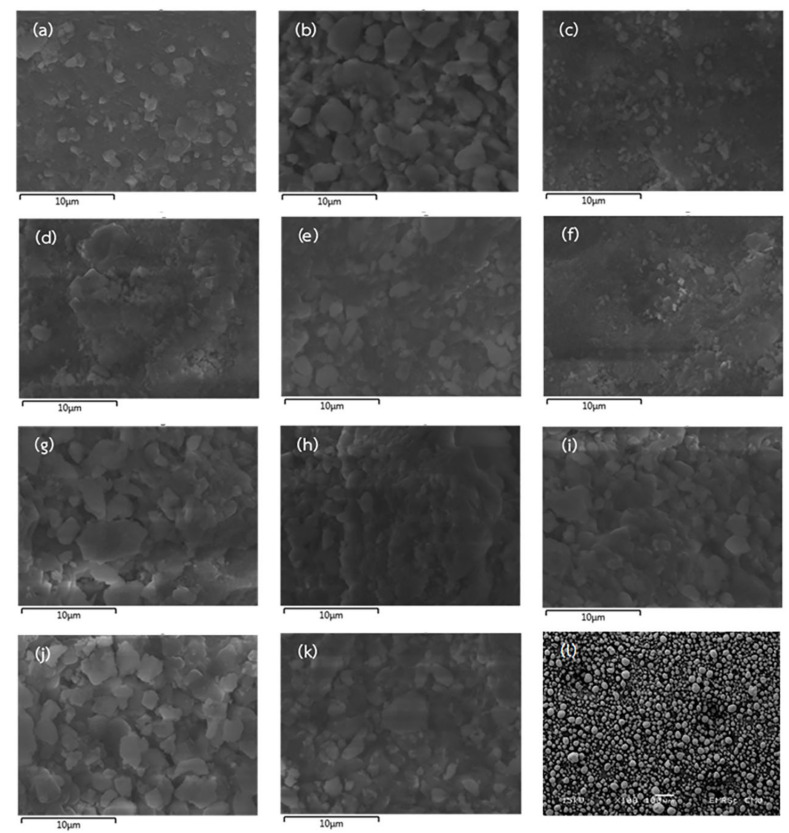
Microstructure from scanning electron microscopy (SEM) of hydroxyapatite obtained by using 2% HCl 10 min (**a**), 5% HCl 10 min (**b**), 2% HCl 60 min (**c**), 5% HCl 60 min (**d**), 2% HCl 35 min (**e**), 5% HCl 35 min (**f**), 3.5% HCl 10 min (**g**), 3.5% HCl 60 min (**h**), 3.5% HCl 35 min Rep.1 (**i**), 3.5% HCl 35 min Rep.2 (**j**), 3.5% HCl 35 min Rep.3 (**k**), and the commercial hydroxyapatite (**l**).

**Figure 4:**
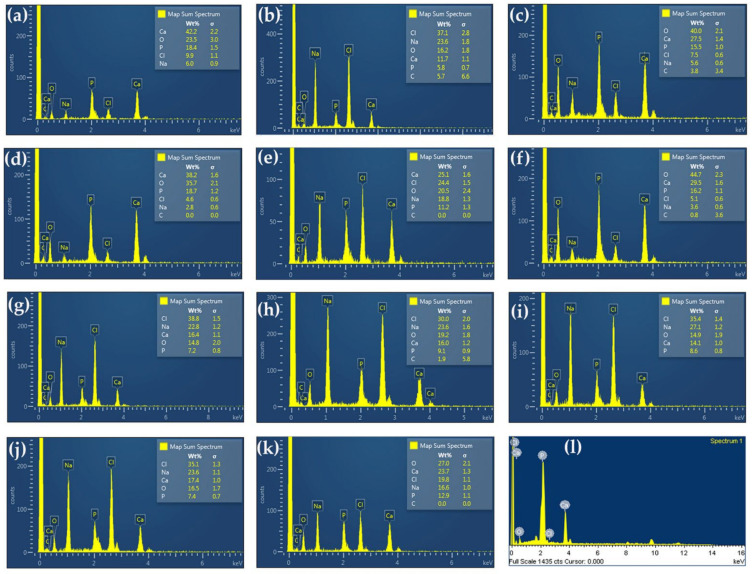
Energy dispersive X-ray (EDX) semiquantitative analysis for elemental estimation for hydroxyapatite extracted using 2% HCl 10 min (**a**), 5% HCl 10 min (**b**), 2% HCl 60 min (**c**), 5% HCl 60 min (**d**), 2% HCl 35 min (**e**), 5% HCl 35 min (**f**), 3.5% HCl 10 min (**g**), 3.5% HCl 60 min (**h**), 3.5% HCl 35 min Rep.1 (**i**), 3.5% HCl 35 min Rep.2 (**j**), 3.5% HCl 35 min Rep.3 (**k**), and the commercial hydroxyapatite (**l**).

The microstructure and estimated Ca and P contents of all prepared and commercial hydroxyapatites were analyzed using the SEM equipped with EDX and shown in Figure 3 and Figure 4, respectively. The commercial hydroxyapatite had numerous agglomerations of small spherical particles, whereas all of the prepared hydroxyapatites had irregular morphology, flat plate, large surface area, roughness, and lower 10 µm of particle size. This was similar to hydroxyapatite obtained from the *Rapana thomasiana* shell, which had irregularly shaped microcrystalline aggregates with a dimension of about 1–10 µm [33]. Therefore, Pon-On et al. [34] reported that the microstructure of extracted hydroxyapatite from fish scales had a flat plate and a large and rough surface.

The EDX results presented the main component of calcium, phosphorus, oxygen, and estimated Ca and P content based on EDX analysis, which was in the range of 1.64–2.35. This agreed with the obtained values from the chemical analysis based on the method of AOAC [35] in Table 1. 

**Figure 5:**
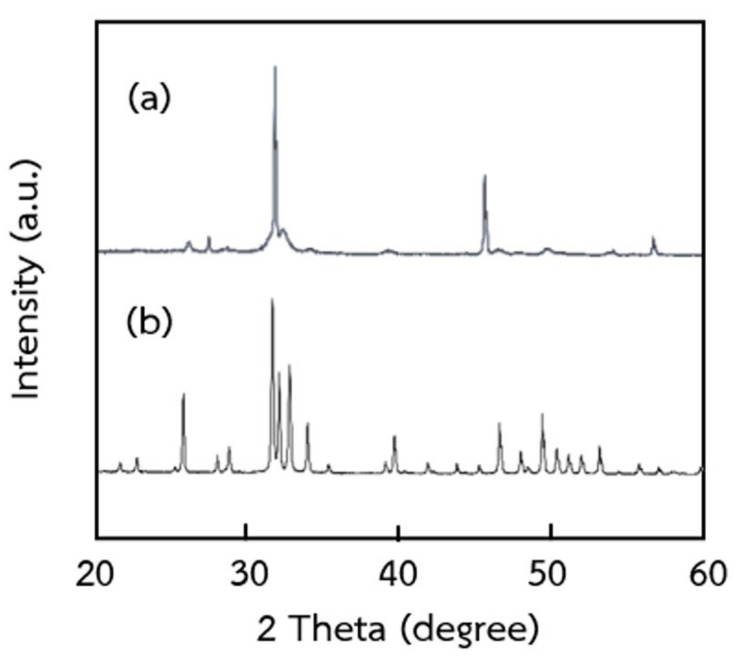
XRD patterns of (**a**) extracted hydroxyapatite using 5% HCl with 60 min extraction time and (**b**) commercial hydroxyapatite.

Based on the optimum conditions, the obtained hydroxyapatite was analyzed by XRD to determine the phase composition. The XRD pattern of extracted and commercial hydroxyapatites is shown in Figure 5. The XRD patterns of extracted hydroxyapatite were the main peaks corresponding to commercial hydroxyapatite. Those XRD patterns were sharp peaks with the highest intensity at 2θ = 31.7°, compatible with the 211 planes of hydroxyapatite [36]. This also was consistent with the X-ray diffraction spectrum of powder and bulk tooth enamel, which showed the intensities of most peaks including (211), (300), (310), and (222) planes [37]. The extracted hydroxyapatite shows the sharp peak of the hydroxyapatite phase, suggesting a high crystallinity of prepared hydroxyapatite. As in previous reports by Nam et al. [8], Pon-On et al. [34], and Panda et al. [36], a complete crystallization of the extracted hydroxyapatite powders was confirmed by the sharp peak intensity of XRD patterns. 

### 2.4. Hydroxyapatite Precipitation from the Extracted Solution

As the obtained optimum condition for hydroxyapatite extraction from bigeye snapper bone, the effects of alkali, and concentration on hydroxyapatite precipitation were determined using NaOH and KOH with various concentrations as shown in Table 4. The obtained hydroxyapatites of all treatments were similar in yield (*p* > 0.05) and slight color differences (*p* ≤ 0.05). The precipitation with KOH obtained slightly higher whiteness hydroxyapatite. However, the main factors for production are yield and cost, and the 5% NaOH was selected to precipitate hydroxyapatite from the extracted solution.

### 2.5. Cell Cytotoxicity

**Figure 6:**
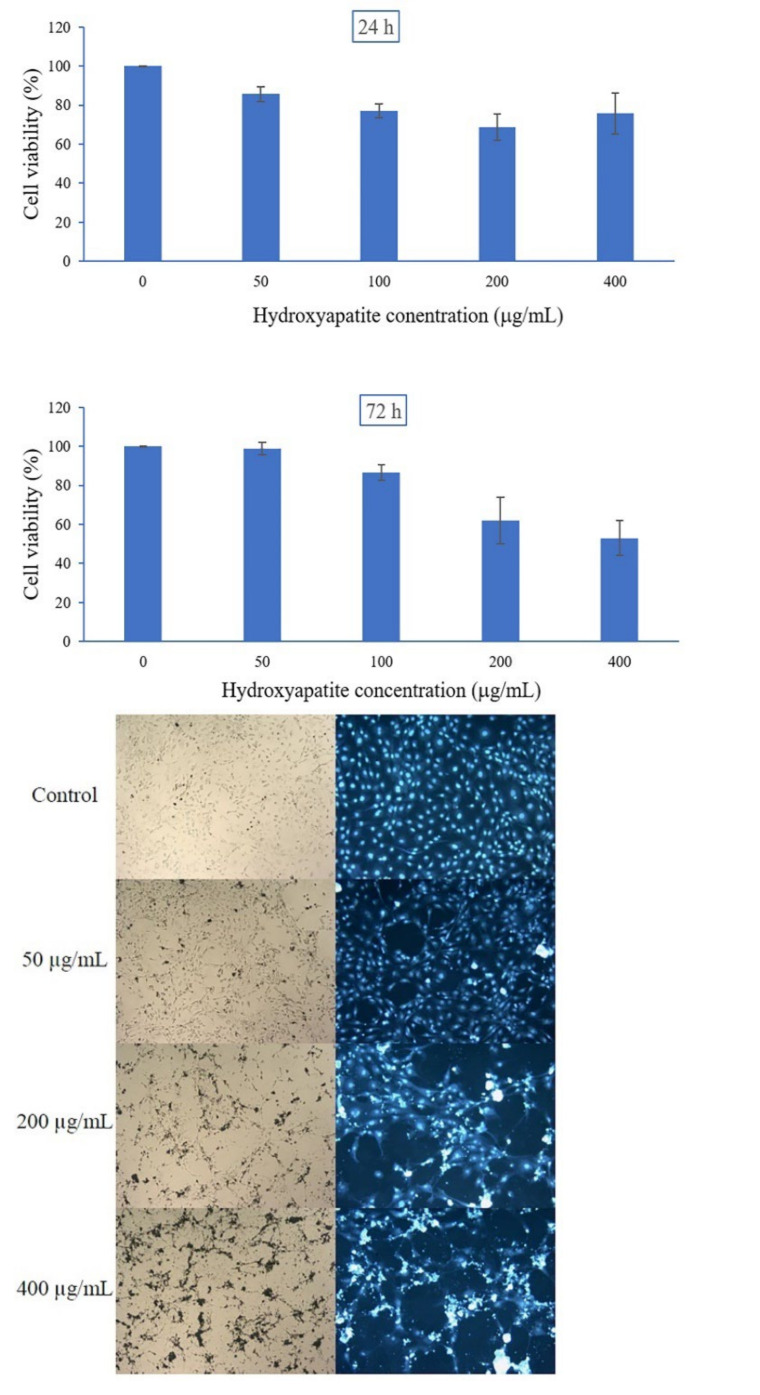
The effects of obtained hydroxyapatite from bigeye snapper bone on MC3T3-E1 cell viability after incubation for 24 and 72 h and cell morphology after incubation for 72 h using optical microscopy and Hoechst stain at 100× original magnifications.

The cytotoxicity on MC3T3-E1 using MTT assay was performed as shown in Figure 6. The extracted hydroxyapatite from bigeye snapper bone was non-cytotoxic and low-cytotoxicity at a concentration of 50 µg/mL and 100–400 μg/mL, respectively. After incubation for 24 and 72 h, cell viability was in the range of 69–86% and 53–99%, respectively. The viability of MC3T3-E1 cells treated with hydroxyapatite was slightly decreased with increasing of concentration. The morphology of the MC3T3-E1 cell line was studied after 72 h treatment of hydroxyapatite as shown in Figure 6. From the optical microscopy and after the Hoechst stain, a slightly decreasing viable cell number was observed when increasing hydroxyapatite concentration. As in previous research, non-cytotoxic extracted hydroxyapatite was reported such as hydroxyapatite from tuna and swordfish bones [29] and hydroxyapatite from rainbow trout and salmon bones, promoting the viability of MC3T3-E1 cells at a concentration of 200 μg/mL [15].

### 2.6. Adsorption of Protein on Hydroxyapatite

**Figure 7:**
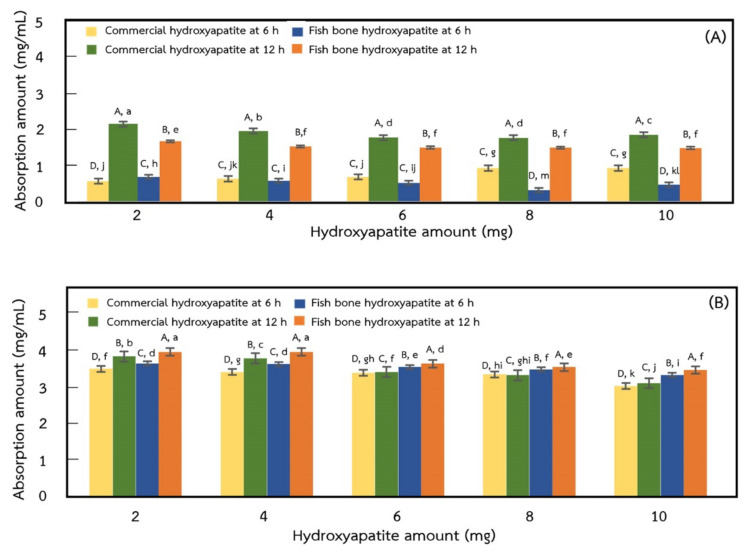
BSA adsorption onto commercial and extracted hydroxyapatites using BSA solutions at concentration of 0.5 mg/mL (**A**) and 1.0 mg/mL for 6 h and 12 h (**B**). Different upper-case letters in same amount of hydroxyapatite indicate significantly differences (*p* ≤ 0.05) and different lower-case letters in all amount of hydroxyapatite indicate significantly differences (*p* ≤ 0.05).

The 3 and 6 mg/mL of BSA solutions were adsorbed on various amounts of commercial and extracted hydroxyapatites for 6 and 12 h, and the results are shown in Figure 7. At 3 mg/mL BSA with 6 h adsorption time, the adsorption amount of BSA on various amounts of commercial and extracted hydroxyapatite (2, 4, 6, 8, and 10 mg) was in the range of 0.56–0.92 mg/mL and 0.44–0.80 mg/mL, respectively. At 3 mg/mL BSA with 12 h adsorption time, the amount of BSA adsorbed on various amounts of commercial and extracted hydroxyapatite (2, 4, 6, 8, and 10 mg) were in the range of 1.87–2.15 mg/mL and 1.49–1.68 mg/mL, respectively. At 6 mg/mL with 6 h adsorption time, the amount of BSA adsorbed on various amounts of commercial and extracted hydroxyapatite (2, 4, 6, 8, and 10 mg) was in the range of 2.99–3.46 mg/mL and 3.30–3.61 mg/mL, respectively, while at 6 mg/mL with 12 h adsorption time, the BSA adsorbed amount on various amounts of commercial and extracted hydroxyapatite (2, 4, 6, 8, and 10 mg) were the range of 3.11–3.84 mg/mL and 3.46–3.95 mg/mL, respectively. These results showed that the protein adsorption of commercial and extracted hydroxyapatites was significantly increased with increasing amounts of hydroxyapatite, adsorption time, and BSA concentration (*p* ≤ 0.05). However, at 3 mg/mL BSA solution with increasing adsorption time from 6 to 12 h, the amount of BSA adsorbed on commercial and extracted hydroxyapatite increased by 2.6 and 2.5 times, respectively, while the amount of BSA adsorbed for 6 mg/mL BSA solution increased about 1.1 times. Adsorbed BSA on hydroxyapatite depends on the hydrophobicity of individual hydroxyapatite and BSA molecules, which enhances their mutual adsorption and interaction behavior [38]. In addition, the electrostatic interaction between cation (Ca^2+^) and anion (PO_4_^3−^) of hydroxyapatite with cation (NH_4_^+^) and anion (COO^−^) of BSA protein influenced the adsorption amount onto hydroxyapatite [39], and the specific surface area of hydroxyapatite generally corresponds to amount of protein adsorption, in which a higher surface area exhibits a higher amount of adsorbed protein [40].

**Figure 8:**
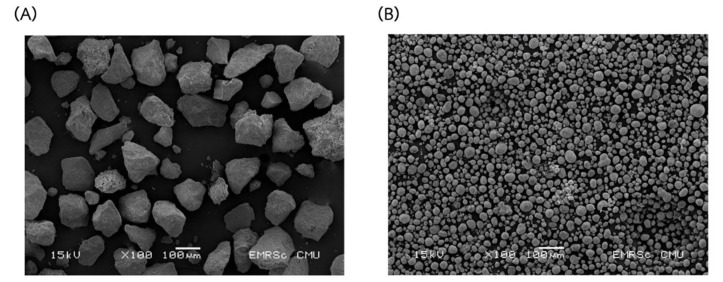
Morphology of extracted (**A**) and commercial hydroxyapatite (**B**) after adsorption of BSA.

**Figure 9:**
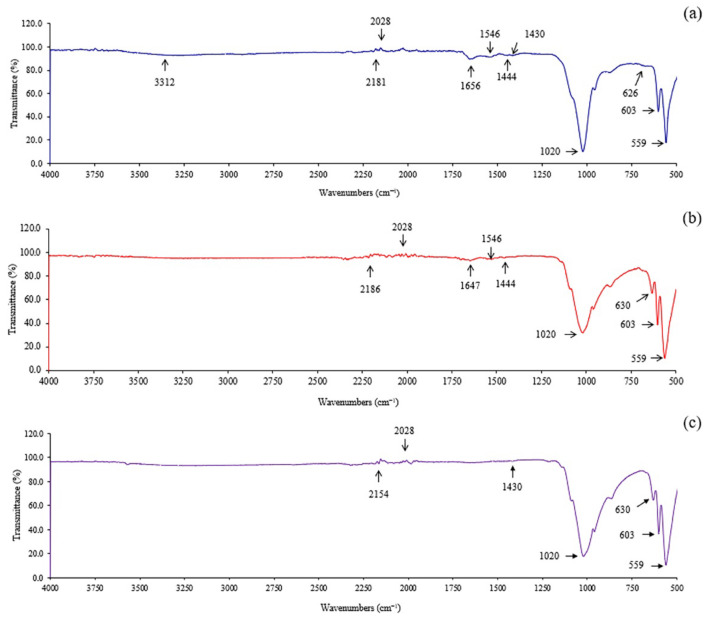
FTIR spectra of extracted (**a**) and commercial hydroxyapatite (**b**) after adsorption of BSA and commercial hydroxyapatite (**c**).

The morphology of hydroxyapatite-adsorbed BSA is shown in Figure 8. From these results, the larger particles and spherical shape of adsorbed particles compared with non-adsorbed BSA (Figure 3) were observed. The presence of BSA in hydroxyapatite was confirmed by FTIR analysis as shown in Figure 9a,b compared with commercial hydroxyapatite (Figure 9c). The bands at 559, 603, and 1020 cm^−1^ are attributed to the phosphate group of hydroxyapatites. In Figure 9a, the BSA adsorbed on the extracted hydroxyapatite showed a vibration band at 3312 cm^−1^ for the formation of a hydroxyl group. The bands at 1656 cm^−1^ and 1546 cm^−1^ are attributed to the N–H bond of amide I and amide II, respectively. The bands at 1444 cm^−1^ and 1546 cm^−1^ indicated the C–H contortion modes of –CH_2_ (methyl) and –CH_3_ (methene) in polypeptides, respectively [41]. In Figure 9b, the BSA adsorbed on commercial hydroxyapatite showed the peaks at 1647, 1546, 1444, and 1546 cm^−1^, which are attributed to amide I, amide II, methyl, and methene, respectively. These results confirmed the amide I, amide II, methyl, and methane of the BSA protein adsorbed onto both hydroxyapatites. 

### 2.7. Desorption of Protein on Hydroxyapatite

**Figure 10:**
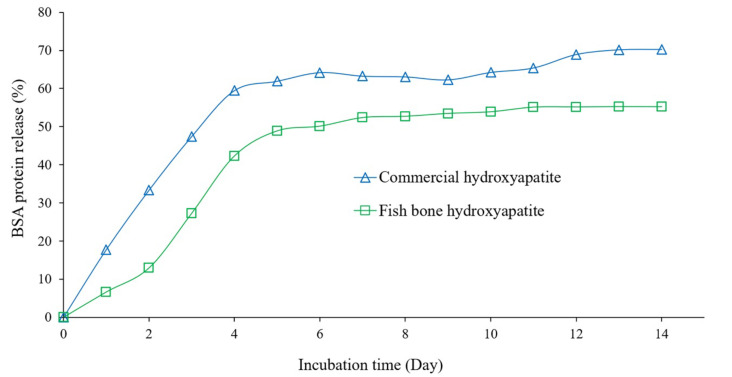
Release amount of the BSA loaded on the commercial and the extracted hydroxyapatite particles at different incubation times.

The protein release behavior of BSA adsorbed on the extracted and the commercial hydroxyapatite in phosphate buffer solution (pH 7.4) at 37 °C was done in different incubation times and shown in Figure 10. The increasing release rate was observed for both hydroxyapatites within the time interval of the first 4 days, and then after day 5, it follows a steady state of the release rate of protein until 14 days. These results are consistent with the research of Swain and Sakar [38], they have reported that the release of the BSA-loaded hydroxyapatite was a rapid release rate in the first 4 days, and then became a steady rate until 10 days. However, in the present work, a higher amount of release protein from the adsorbed BSA on commercial hydroxyapatite was observed, and the highest release amount for the BSA loaded on commercial and extracted hydroxyapatite was 70% and 55%, respectively. This might be due to the resulting smaller particles and spherical shape of the commercial hydroxyapatite compared with the irregular shape of extracted hydroxyapatite particles (Figure 8). The previous report of Bharath et al. [42] found that the release rate depends on the pH of the used solutions, pH 4.4 resulted in a higher release rate than pH 7.4. Therefore, a high adsorption capacity along with slow and sustained release behavior of hydroxyapatite particles might be a promising candidate material for protein carrier systems with a controlled release [20,37,42].

## 3. Materials and Methods

### 3.1. Material and Preparation 

The bones of bigeye snapper (*Priancanthus tayenus*) were a byproduct of fish mince processing, which were obtained from Sirikhun Seafood Co., Ltd. (Samut Sakhon, Thailand). Fish bones were packed into polyethylene bags (5 kg/bag), frozen, and transported to the laboratory of the College of Maritime Studies and Management, Chiang Mai University (Samut Sakhon) using a container car (temperature −18 to −20 °C). On arrival, the frozen fish bones were thawed, washed, and crashed using a silent cutter (YS07, kitchen mall, Pathum Thani, Thailand), packed into zip lock polyethylene bags (1000 g/bag), and stored at −18 to −20 °C until further use (not over than 2 months). 

### 3.2. Chemicals

Sodium hydroxide (NaOH), hydrochloric acid (HCl), sulfuric acid (H_2_SO_4_), and potassium hydroxide (KOH) were purchased from Merck (Billerica, Germany). Hydroxyapatite (calcium phosphate tribasic) and bovine serum albumin (BSA) were purchased from Sigma-Aldrich (St. Louis, MO, USA). Other chemicals and reagents used were analytical grade.

### 3.3. Hydroxyapatite Extraction from Bigeye Snapper Bones

The hydroxyapatite extraction from bigeye snapper bones was carried out according to the method of Pon-On et al. (2016) with a slight modification. The frozen crushed fish bones were thawed at 5–7 °C overnight (~10 h) before use as a raw material. The 500 g of fish bones were soaked in an HCl solution (2–5% *w*/*v*) with a 1:2 (*w*/*v*) of fish bone: acid solution ratio several times (10–60 min). Afterward, the extracted hydroxyapatite solution was obtained by filtration with a double layer of cheesecloth. Then the hydroxyapatite-rich slurry was obtained by adding the 10% NaOH solution until pH became 7, filtered using filter paper (Whatman No. 1) to keep the solid part (the extracted hydroxyapatite cake), and rinsed with deionized water. The obtained hydroxyapatite cake was packed into a plastic bag and heated at 95°C for 45 min using a water bath (WNB45, Memmert, Germany), and dried at 60°C using a hot air oven (FD260, Binder, Germany) for 24 h. Dried hydroxyapatite was ground using a blender (BlendforceBL478, Tefal, Bangkok, Thailand) and sieved with a 150 mesh sieve to obtain a fine powder.

### 3.4. Optimization of Experimental Design 

The optimum conditions for the preparation of hydroxyapatite from bigeye snapper bones were determined using response surface methodology (RSM) with a faced-centered central composite design. The effects of extraction time (X_1_: 10–60 min) and concentration of HCl (X_2_: 2–5% *w*/*v*) on the yield, calcium (Ca) and phosphorus (P) content, and Ca/P ratio of the obtained hydroxyapatite were investigated. The 11 experiment units were generated as shown in Table 1. Design Expert software (Version 11, Stat-Ease, Inc., Minneapolis, MN, USA) was used for the design of the experiment, analysis, and response surface plots. A full quadratic model for each response was obtained and expressed with real variables using the following equation:

Y_i_ = β_0_ + β_1_X_1_ + β_2_X_2_ + β_11_X_1_^2^ + β_22_X_2_^2^ + β_12_X_1_ X_2_

where Yi is the response variables, and X_1_ and X_2_ are the independent variables, β_0_ = the coefficients for the constant, β_1_ and β_2_ = the coefficients for the linear terms, β_11_ and β_22_ = the coefficients for the quadratic terms, and β_12_ = the coefficients for the interaction terms. 

### 3.5. Analyzes

#### 3.5.1. Proximate Compositions

Moisture, protein, lipid, and ash contents in the fresh bigeye snapper bone were determined according to the AOAC methods [43]. 

#### 3.5.2. Hydroxyapatite Extraction Yield

The yields of hydroxyapatite extracted from bigeye snapper bone were calculated using the following equation:

$$\mathrm{Yield}\;\left(\%\right)=\frac{\mathrm{Dried}\;\mathrm{hydroxyapatite}\;\mathrm{weight}\;\left(g\right)}{\mathrm{bigeye}\;\mathrm{snapper}\;\mathrm{bones}\;\mathrm{used}\;\mathrm{for}\;\mathrm{extraction}\;\left(g\right)}\times 100$$

#### 3.5.3. Color Determination

Hydroxyapatite color (L*, a*, b* values) was determined using a Hunter Lab Colorimeter (ColorFlex EZ Spectrophotometer, VA, USA). The color difference (Δ*E*) was calculated using the following equation:

Δ*E* = [(ΔL*)^2^ + (Δa*)^2^ + (Δb*)^2^]^1/2^

where ΔL*, Δa*, and Δb* are the difference between the color parameters of samples and the white standard plate (91.40 of L*, −1.37 of a*, −0.33 of b*).

#### 3.5.4. Calcium and Phosphorus Content

The calcium and phosphorus contents were determined according to the 984.27 of the AOAC method [35]. In brief, 1–2 g of hydroxyapatite was placed in the hydrolysis bottle, 10 mL of the nitric acid and perchloric acid mixture (2:1 *v*/*v*) was added, incubated at room temperature for 1 h, and digested in the digestion block (block digestion system DG-U-A021, Seal Analytical, WI, USA). The volume of digested sample was adjusted to 50 mL using a volumetric flask by adding deionized water. Then the calcium and phosphorus contents were analyzed using an inductively coupled plasma emission spectrometer (ICP-OES) (Optima 8000, PerkinElmer, OH, USA). The calcium or phosphorus contents were reported in the g/100 g sample. The calcium/phosphorus ratio (Ca/P ratio) was calculated based on those contents. 

#### 3.5.5. Fourier Transform Infrared Spectroscopy (FTIR)

FTIR spectra of hydroxyapatites were obtained using an attenuated total reflection Fourier transform infrared (ATR-FTIR) spectrometer (NICOLET 6700FT-IR, Thermo Fisher Scientific, MA, USA). The spectra were recorded at a resolution of 4000–400 cm^−1^ with a resolution of 4 cm^−1^.

#### 3.5.6. Scanning Electron Microscope (SEM) and Energy Dispersive X-ray (EDX)

The morphology of hydroxyapatite powder was observed using low vacuum SEM equipped with an EDX analyzer (JSM 5910 LV, JEOL, Tokyo, Japan) to estimate local mineral content. The hydroxyapatite powder was glued on conductive carbon adhesive tapes struck with the SEM stub and coated with gold using an automatic fine coater (JFC1600; JEOL Ltd.). The workpiece was subjected to the specimen chamber with 12.5 mm between the camera and the workpiece and the beam spot size 30, and then we obtained the microstructure.

#### 3.5.7. X-ray Diffraction Analysis (XRD)

The phase composition of the extracted compounds of hydroxyapatite was obtained using X-ray diffraction (Miniflex II, Rigaku, Japan). The XRD patterns of hydroxyapatite were obtained. The measurement was done at a voltage of 40 kV and 40 mA in 2θ angles from 20–100 degrees, measuring every 0.02 degrees at a speed of 0.4 s/time. 

### 3.6. The Precipitation of Hydroxyapatite from the Extracted Solution

The NaOH and KOH solutions (concentrations of 5, 10, 15, and 20% *w*/*v*) were studied to obtain the optimum condition for hydroxyapatite precipitation from the extracted solutions. The hydroxyapatite extracted solution was prepared using the optimum condition detailed in Section 2.2 and precipitated by adding a different alkali solution with various concentrations until the pH of the solution reached a natural pH of 7. Then hydroxyapatite was separated in the same manner as previously mentioned in Section 3.3, and the hydroxyapatite yield was calculated.

### 3.7. In Vitro Cell Viability

Mouse calvaria cells (MC3T3-E1 subclone 14 cell line) were purchased from Elabscience Biotechnology Inc. (TX, USA). MC3T3-E1 cells were cultured in alpha minimum essential medium (α-MEM) supplemented with 10% heat-inactivated fetal bovine serum (FBS), 2 mM glutamine, and 1% penicillin (100 U/mL) in a humidified atmosphere with 5% CO_2_ at 37 °C. The effects of hydroxyapatite on MC3T3-E1 cell viability were measured via the reduction of 3-(4,5-dimethyl-thiazol-2yl)-2,5-diphenyl tetrazolium bromide (MTT) colorimetric assay. Briefly, MC3T3-E1 cells were seeded on a 96-well culture plate at 1 × 10^4^ cells/well (100 µL) and were incubated for 24 h to obtain cells that were attached to the substratum. The cells were treated with hydroxyapatite at various concentrations (50, 100, 200, and 400 µg/mL), and some were left untreated (control) for 24 and 72 h at 37 °C. After incubation, 15 µL of the MTT solution (5 mg/mL in phosphate-buffered saline; PBS) was added to each well. After subsequent incubation for 4 h, the purple-colored precipitates were obvious. The supernatant was removed, and the formazan precipitates were solubilized with the addition of 100 µL DMSO per well. Then, after 10 min of incubation, the optical density (OD) was obtained at 540 and 630 nm using a scanning multi-well microplate reader (SpectraMax i3x, Molecular Devices, CA, USA). Three replicate samples were tested for each condition. The cell viability was calculated using the following equation:

% Cell viability = (OD_T_ /OD_C_) × 100

where OD_T_ is OD of treated at 540 nm—OD_C_ of treated at 630 nm and OD_C_ is OD of untreated (control) at 540 nm—OD of untreated (control) at 630 nm 

Cell morphology was studied after a treatment period of 72 h. The MC3T3-E1 cells were stained with 50 µg/mL Hoechst 33258 and incubated at 37 °C for 20–30 min in darkness. After washing with PBS three times, the cell morphology was observed under a fluorescence microscope (BDS300-PH series; DRAWELL, Shanghai, China) at 100× original magnifications and the images were recorded.

### 3.8. Adsorption of Protein on Hydroxyapatite

Protein adsorption on hydroxyapatite was measured according to the method of Kojima et al. (2018) with a slight modification. The BSA with concentrations of 3 and 6 mg/mL in a 10 mmol/L phosphate buffer solution (pH 7.0) was prepared. Then, different amounts of hydroxyapatite (2, 4, 6, 8, 10 mg) were added to 1 mL of the prepared BSA solution. The mixture was stirred at 20 °C for 6 and 12 h and then centrifuged (Frontier™ 5718R, Ohaus Corporation, Parsippany, NJ, USA) at 4400× *g* for 5 min to collect the supernatant. The excess protein in the supernatant was estimated using the Bradford method by a microplate reader (Varioskan Lux microplate reader, Thermo Fisher Scientific Inc., Singapore) at λ = 595 nm. The amount of protein adsorption was calculated in accordance with the difference between the initial and excess protein concentrations.

### 3.9. Desorption of Protein from Protein-Adsorbed Hydroxyapatite

Desorption of protein from protein-adsorbed hydroxyapatite was investigated according to the method of Tomoda et al. [17] with a slight modification. Hydroxyapatite absorbing BSA particles (4 mg) were dissolved in 1 mL of 10 mmol/L phosphate buffer solution (pH 7.4) and incubated at 37 °C for 1–14 days. After predetermined incubation times, the mixtures were centrifuged (Frontier™ 5718R, Ohaus Corporation) at 4400× *g* for 10 min to collect the supernatant. The absorbance of the supernatant was obtained at 280 nm using a microplate reader (Varioskan Lux microplate reader).

### 3.10. Statistical Analysis

One-way analysis of variance (ANOVA) and Duncan’s new multiple range tests (DMRT) were performed for the significant differences between means (*p* ≤ 0.05) using the Statistical Package for the Social Sciences, SPSS statistical package 17.0 software (SPSS Inc., Chicago, IL, USA). All experiments and data are presented as the mean ± standard deviation of three replications.

## 4. Conclusions

The optimum condition for the preparation of hydroxyapatite from bigeye snapper bone was the extraction with 5% HCl for 60 min, which both factors of HCl concentration and extraction time were the main effect for the hydroxyapatite yield and color. The obtained hydroxyapatite yield and Ca/P ratio were 13.4% w and 2.1, respectively. The 5% NaOH solution was the optimum for hydroxyapatite precipitation from the extracted solution. The microstructure of prepared hydroxyapatite was irregular morphology, flat plate, large surface area, and roughness. The XRD pattern and FTIR spectra confirmed the composition of the extracted hydroxyapatite compared with commercial hydroxyapatite. Non- and low-cytotoxicity was found at the extracted hydroxyapatite concentration of 50 and 100–400 μg/mL, respectively. The BSA protein adsorption capacity of extracted hydroxyapatite was significantly increased with increasing hydroxyapatite amount, adsorption time, and BSA concentration. The protein release from BSA loaded on hydroxyapatite was increased initially in the first 4 days and become a steady release rate until 14 days. This study suggested possibilities to utilize bigeye snapper bone as a good material for the preparation of natural hydroxyapatite, which may lead to it being a beneficial material in protein delivery systems.

## Figures and Tables

**Table 1:** Experimental unit and response variables for hydroxyapatite extraction from bigeye snapper bones.

Treatment	X_1_ (min)	X_2_ (%)	Yield (%)	Ca ^ns^ (g/100 g)	P ^ns^ (g/100 g)	Ca/P Ratio ^ns^	L*	a*	b*	Δ*E*
1	10	2.0	4.26 ± 0.20 ^e^	15.0 ± 4.87	7.38 ± 2.24	2.03 ± 0.03	79.8 ± 0.13 ^e^	4.16 ± 0.38 ^a^	19.3 ± 0.91 ^a^	31.1 ± 1.24 ^a^
2	10	5.0	10.6 ± 0.16 ^bc^	19.7 ± 1.58	9.43 ± 1.12	2.09 ± 0.06	90.3 ± 2.83 ^ab^	4.20 ± 1.63 ^a^	20.3 ± 2.18 ^a^	21.8 ± 1.89 ^c^
3	60	2.0	6.77 ± 0.43 ^d^	16.4 ± 2.44	8.08 ± 1.23	2.03 ± 0.00	75.2 ± 0.76 ^e^	3.62 ± 0.78 ^ab^	18.1 ± 1.33 ^ab^	29.0 ± 2.05 ^ab^
4	60	5.0	13.4 ± 1.30 ^a^	19.8 ± 3.90	9.55 ± 2.07	2.08 ± 0.03	91.3 ± 1.35 ^a^	2.84 ± 0.80 ^c^	16.5 ± 2.14 ^bc^	11.1 ± 2.39 ^f^
5	35	2.0	6.25 ± 0.11 ^d^	17.9 ± 0.03	8.65 ± 0.03	2.07 ± 0.00	76.9 ± 2.01 ^e^	3.11 ± 0.82 ^b^	18.6 ± 1.94 ^ab^	30.6 ± 0.67 ^e^
6	35	5.0	12.4 ± 1.03 ^a^	17.2 ± 2.58	8.28 ± 1.21	2.08 ± 0.00	89.7 ± 0.87 ^ab^	2.67 ± 0.29 ^c^	17.4 ± 1.42 ^b^	16.1 ± 0.43 ^b^
7	10	3.5	9.26 ± 0.15 ^c^	17.5 ± 1.16	8.30 ± 0.56	2.11 ± 0.00	87.1 ± 1.09 ^abc^	2.88 ± 0.25 ^c^	17.1 ± 1.57 ^b^	28.8 ± 1.57 ^d^
8	60	3.5	11.8 ± 0.71 ^ab^	20.1 ± 0.59	9.52 ± 0.39	2.11 ± 0.02	77.9 ± 2.55 ^de^	2.47 ± 0.23 ^c^	17.2 ± 0.73 ^b^	18.4 ± 1.31 ^c^
9	35	3.5	9.42 ± 0.12 ^c^	19.7 ± 0.80	9.27 ± 0.70	2.13 ± 0.05	85.6 ± 0.62 ^bc^	1.04 ± 0.35 ^d^	11.7 ± 2.55 ^d^	22.6 ± 0.28 ^c^
10	35	3.5	10.3 ± 0.15 ^bc^	16.2 ± 0.14	7.83 ± 0.30	2.06 ± 0.04	87.7 ± 0.62 ^abc^	1.05 ± 0.59 ^d^	10.8 ± 1.45 ^e^	22.9 ± 0.39 ^c^
11	35	3.5	10.4 ± 0.21 ^bc^	19.2 ± 2.24	9.23 ± 1.10	2.08 ± 0.00	84.4 ± 1.65 ^cd^	1.68 ± 0.44 ^d^	12.7 ± 1.49 ^d^	22.2 ± 2.00 ^c^

**Note**: X_1_: Extraction time (min), X_2_: HCl concentration (%). Different superscript letters in the same column indicate statistical differences (*p* ≤ 0.05) and ^ns^ is not a significant difference (*p* > 0.05).

**Table 2:** Quadratic models of hydroxyapatite preparation.

Responses	Quadratic Polynomial Model	R^2^	*p*-Value
Yield (%)	Y_1_ = −5.82 + 6.01X_1_ + 0.05X_2_ − 0.002X_1_X_2_ − 0.57X_1_^2^ − 0.0001X_2_^2^	0.9827	0.0002
L*	Y_2_ = 81.3 + 1.59X_1_ + 0.05X_2_ − 0.012X_1_X_2_ − 0.03X_1_^2^ + 0.00003X_2_^2^	0.9000	0.0154
a*	Y_3_ = 6.10 − 0.77X_1_ − 0.02X_2_ + 0.008X_1_X_2_ − 0.06X_1_^2^ − 0.0002X_2_^2^	0.9741	0.0006
b*	Y_4_ = 17.5 + 2.44X_1_ + 0.004X_2_ + 0.01X_1_X_2_ − 0.78X_1_^2^ − 0.001X_2_^2^	0.9512	0.0027
Δ*E*	Y_5_ = 45.2 − 14.8X_1_ − 0.43X_2_ − 0.02X_1_X_2_ + 2.165X_1_^2^ + 0.01X_2_^2^	0.8772	0.0250
Ca content (g/100 g)	Y_6_ = 18.41 + 1.24X_1_ − 0.68X_2_ − 0.3X_1_X_2_ − 0.94X_1_^2^ + 0.29X_2_^2^	0.4770	0.5382
P content (g/100 g)	Y_7_ = 8.77 − 0.53X_1_ + 0.34X_2_ − 0.15X_1_X_2_ − 0.31X_1_^2^ + 0.14X_2_^2^	0.4653	0.5587
Ca/P ratio	Y_8_ = 2.10 + 0.02X_1_ − 0.002X_2_ − 0.003X_1_X_2_ − 0.035X_1_^2^ + 0.0003X_2_^2^	0.5782	0.3688

**Note**: X_1_: extraction time (min), X_2_: HCl concentration (%), Y_1_: %Yield, Y_2_: L*, Y_3_: a*, Y_4_: b*, Y_5_: Δ*E*, Y_6_; Ca content (g/100 g), Y_7_: P content (g/100 g), Y_8_: Ca/P ratio.

**Table 3:** Criteria of parameters for multi-response optimization, optimum condition, composite desirability, predicated value, and experimental value from the verification of optimum model for yield, L*, a*, b*, and Δ*E*.

Response & Factors	Parameter					Predicated Value	Experimental Value	Composite Desirability
	Goal	Lower	Upper	Weight	Importance			
X_1_	Is in range	10	60	1	3	60	60	0.908
X_2_	Is in range	2	5	1	3	5	5	
Yield (%)	maximize	4.26	13.39	1	3	13.39	13.4 ± 1.30	
L*	maximize	84.96	88.65	1	3	88.0	84.5 ± 2.21	
a*	Is in range	1.03	4.20	1	3	1.47	2.84 ± 0.80	
b*	Is in range	10.83	20.32	1	3	12.25	16.5 ± 2.14	
Δ*E*	Is in range	8.25	20.39	1	3	16.31	15.6 ± 2.95	
Ca content (g/100 g)	none	15.03	20.07	1	3	19.31	19.8 ± 3.90	
P content (g/100 g)	none	7.38	9.55	1	3	9.29	9.55 ± 2.07	
Ca/P ratio	none	2.03	2.13	1	3	2.08	2.08 ± 0.03	

**Note:** X_1_: extraction time (min), X_2_: HCl concentration (%).

**Table 4:** The effects of alkali and concentration on hydroxyapatite precipitation.

Treatment	Yield ^ns^ (%)	L*	a*	b*	*∆*E
5% NaOH	15.89 ± 1.22	85.84 ± 0.01 ^f^	2.81 ± 0.01 ^c^	15.10 ± 0.00 ^c^	16.76 ± 0.00 ^c^
10% NaOH	15.93 ± 1.06	87.56 ± 0.01 ^d^	2.33 ± 0.01 ^e^	14.31 ± 0.02 ^e^	15.05 ± 0.01 ^e^
15% NaOH	16.08 ± 0.30	86.59 ± 0.01 ^e^	2.56 ± 0.01 ^d^	14.40 ± 0.05 ^d^	15.78 ± 0.02 ^d^
20% NaOH	16.09 ± 1.10	84.36 ± 0.03 ^h^	3.30 ± 0.00 ^a^	16.99 ± 0.05 ^b^	18.98 ± 0.04 ^a^
5% KOH	15.11 ± 1.19	88.50 ± 0.02 ^b^	1.76 ± 0.00 ^h^	13.60 ± 0.03 ^g^	13.91 ± 0.03 ^g^
10% KOH	16.08 ± 0.39	88.78 ± 0.03 ^a^	1.96 ± 0.01 ^g^	13.98 ± 0.03 ^f^	14.06 ± 0.02 ^f^
15% KOH	16.87 ± 0.24	88.06 ± 0.01 ^c^	2.02 ± 0.01 ^f^	13.38 ± 0.02 ^h^	14.08 ± 0.02 ^f^
20% KOH	17.05 ± 1.32	85.23 ± 0.01 ^g^	3.08 ± 0.01 ^b^	17.27 ± 0.02 ^a^	18.50 ± 0.02 ^b^

**Note:** Mean ± SD, Different letters in same column indicate significantly differences (*p* ≤ 0.05) and ^ns^ is not a significant difference (*p* > 0.05).

## Data Availability

Data are reported in the article.
